# The impact of public fiscal expenditure on industrial transformation and upgrading: An inverted U-shape evidence from China

**DOI:** 10.1016/j.heliyon.2024.e38456

**Published:** 2024-09-26

**Authors:** Junfeng Zhao, Jinling Yan

**Affiliations:** a College of Economics, Xinjiang University of Finance and Economics, Urumqi, 830012, China; b China (Xinjiang) and Central Asia Regional Economic Cooperation Research Center, Urumqi, 830012, China; c College of International Business and Economics, Xinjiang University of Finance and Economics, Urumqi, 830012, China

**Keywords:** Public fiscal expenditure, Industrial transformation and upgrading, Spatial Durbin model, Inverted U-Shape

## Abstract

High-quality economic development relies on industrial transformation and upgrading. To promote industrial transformation and upgrading, efficient fiscal expenditures are undoubtedly important as pillars of national governance. However, in the context of the market economy, the government's excessive intervention in industrial development will lead to the "promotion tournament" of officials and the "beggar-thy-neighbor" local protectionism, resulting in the convergence of regional industrial structure, which will bring uncertain impact on the upgrading of regional industrial structure. Thus, this study empirically assesses how public fiscal expenditure impacts industrial transformation and upgrading as well as the mechanism by developing a spatial econometric model using the panel data of 250 Chinese cities from 2007 to 2020 and further discusses the differential impact from the perspective of urban scale. The findings disclose that public fiscal expenditure serves a crucial role in facilitating industrial transformation and upgrading, but their relationship resembles an inverted U. Therefore, an optimal scale of public fiscal expenditure exists. Heterogeneity findings reveal that the promoting effect of public fiscal expenditure on industrial transformation and upgrading decreases with the expansion of the city scale. The role mechanism implies that public fiscal expenditure indirectly leverages industrial transformation and upgrading through promoting technological innovation, reducing resource dependence, and expanding scale economies. The conclusion provides a theoretical and practical framework for the government to optimize public fiscal expenditure, promote the transformation and upgrading of China's industrial structure, and ultimately attain high-quality development.

## 1 Introduction

Globally, profound changes are taking place today. New waves of scientific and technological revolution and industrial transformation are underway, as well as fundamental changes in the global industrial structure[1,2]. With the energy crisis and ecological runaway evolution in the late 1960s, the green movement came into being, which has since opened the era of the green economy [3]. Green development is an important concept affecting the overall development of a country. It is an inevitable choice to break through the resource and environmental bottlenecks, transform the development mode, and achieve sustainable development. Both developed countries and emerging economies are facing the urgent task of industrial transformation and upgrading. Based on the need for ecological protection or sustainable development, countries have carried out the “green” adjustment of industrial structure. Green development and green transformation have become a huge trend, which has penetrated all fields of social life and has been widely used [4]. A new wave of change, transformation, and adjustment is sweeping the world. It can be said that in the new era of globalization, industrial transformation, and upgrading have become a common topic of the world.

**Fig. 1:**
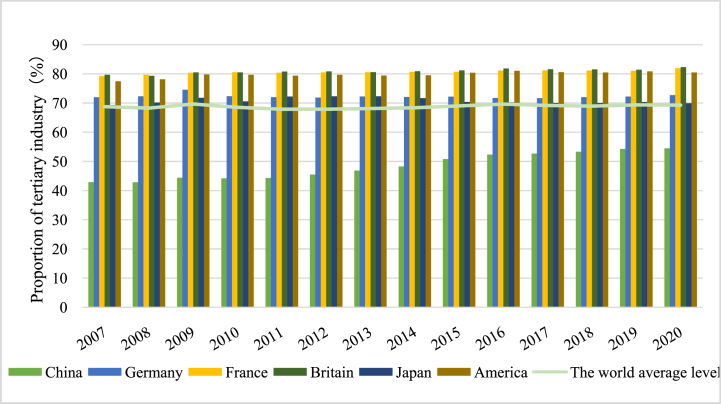
The proportion of tertiary industry.

Industrial transformation and upgrading are becoming a vital tool for increasing the global influence of all countries [5]. As of now, the proportion of China's tertiary industry is far lower than the world average, especially lower than some developed countries in Europe and America (see Fig. 1). China still faces the challenging task of industrial transformation and upgrading. The 20th National Congress of the Communist Party of China emphasized the need for rapid green transformation of the development mode so that high-quality development can be met. The key to green transformation is to change the past extensive development mode and boost industrial transformation and upgrading [6,7]. Industrial transformation and upgrading is not only an essential requirement of China's economic green transformation but also the main channel for high-quality economic development. However, the traditional resource-consuming industries with low added value such as coal and steel have triggered overcapacity and serious pollution, whereas high-tech industries, green industries, and other high-added value industries make up a relatively small percentage of the industrial structure, which seriously restricts China's industrial transformation and upgrading [8]. Practical experience shows that relying only on market mechanisms to guide the industrial transformation may be inefficient [[9], [10], [11]]. As an important pillar of national governance, public finance expenditures substantially contribute to industrial transformation and upgrading [12]. However, the implementation of reducing taxes and fees will bring continuous pressure on fiscal expenditure.

As a result, how to improve the efficiency of fiscal funds in guiding industrial transformation and upgrading is crucial for achieving economic growth in a high-quality manner. The following issues are the research targets of this paper: Can public fiscal expenditure promote industrial transformation and upgrading towards green and efficient development? What is its internal mechanism? What is the heterogeneous impact of different city sizes on the relationship between them? In conclusion, this study represents a theoretical and practical foundation for optimizing public fiscal expenditures, promoting industrial transformation and upgrading in China, and ultimately attaining high-quality development.

Marginal contributions in three areas are made in this study: Firstly, using the super efficiency EBM model, we calculate industrial transformation and upgrading level, which takes unexpected output into account. Compared with the traditional method, such calculation is more scientific and comprehensive. Secondly, this paper brings public fiscal expenditure and industrial transformation and upgrading into the same research framework, and finds out how public fiscal expenditure impacts industrial transformation and upgrading by spatial econometric model. Thirdly, the nonlinear relationship between public fiscal expenditure and industrial transformation and upgrading as well as the influencing mechanism between them is further explored.

This paper continues by presenting the following sections: in Section 2, an analysis of the relevant literature is conducted; the mechanism and theoretical hypothesis are presented in Section 3; the data and model framework is demonstrated in Section 4; Section 5 presents empirical tests and the discussion of results. Conclusions, policy implications, and future research recommendations are discussed in the concluding section.

## 2 An overview of the literature

Public fiscal expenditure is the macro-control tool of government and a vital influencing factor in industrial transformation and upgrading[11,13]. Scholars have studied how public fiscal expenditure impacts industrial transformation by adopting different methods and data samples, and the conclusions are divided. The following mainly sort out the relevant studies on them.

Most scholars believe that the scale of public fiscal expenditure can make up for the lack of market mechanisms in upgrading the industrial structure. However, some scholars pointed out that public fiscal expenditure cannot promote industrial transformation and upgrading, instead, it may hinder the optimization of industrial structure.

Ref. [14] proposed to optimize and upgrade industrial structure by increasing government intervention to guide the direction of demand and improve the demand structure. According to Ref. [3], polluting enterprises will become more energy efficient, and reduce their energy use by increasing environmental regulation or taxes, thus leading to changes in industrial structure. The study by Ref.[11,15] examined how public fiscal expenditure impacts secondary industry transformation and upgrading, and found that public fiscal expenditure is capable of enhancing industrial upgrading by transforming traditional industries, supporting pillar industries, and cultivating emerging industries through direct expenditure and monetary policy. Ref. [16]analyzed the impact of the scale and structure of local public fiscal expenditure on China's industrial transformation and upgrading using a spatial econometric model, finding a significant spatial spillover effect of public fiscal expenditures on the upgrading of industrial structure. Fiscal expenditure can promote the optimization of industrial structure, and its transmission mechanism lies in the fact that the increase of fiscal expenditure will enhance the R&D willingness of enterprises, improve the initiative of product innovation, and then increase the proportion of emerging industries in the overall industry, to effectively promote the upgrading of industrial structure[[11], [13], [14], [15], [16], [17]]. In addition, increasing fiscal expenditure, especially expenditure on science, education, and culture, can improve the overall quality of the labor force, and the improvement of human capital is the key to promoting the transformation and upgrading of economic structure [18].

In contrast, others assert that public fiscal expenditure will cause excessive intervention in the market, which inhibits industrial upgrading [19,20]. As a result of administrative intervention, local officials are pushed into "promotion championships" and local protectionism emerges, resulting in the convergence of regional industrial structures [21]. At present, China's industrial structure is heavily influenced by the local government's preference for attracting investment and the market's impulse to invest [22,23]. Ref. [24] believe that due to the “promotion tournament”, there is a tendency for local governments to focus excessively on GDP and to invest fiscal expenditures in the secondary industry that offers quick and good economic returns. However, the secondary industry is dominated by manufacturing but also includes extractive industries, electricity, and gas industries, which are all pollution-intensive industries, thus inhibiting industrial upgrading. Additionally, more fiscal expenditure flows to “large traditional advantageous enterprises” or to infrastructure industries that promote economic development, while emerging technology enterprises that are conducive to the optimization of industrial structure receive less financial support, which will hinder the upgrading of industrial structure [25].

Closely related to this paper are the studies on the impact of fiscal expenditure on green growth and technology innovation. Scholars have different views on the relationship between fiscal expenditure and green growth. One view is that fiscal spending can achieve both economic and environmental effects. Government investment in environmental governance has a significant impact on the green economy, and the increase in the proportion of total fiscal expenditure on environmental protection has a positive promoting effect on green technological progress and green economic growth [26]. Another view is that the economic effect and environmental effects brought by the government's environmental protection investment are different in intensity [27]. Scholars also hold different views on the impact of government spending on technological innovation. Most of them believe that financial subsidies, as a direct income of enterprises, can increase the cash flow of enterprises, reduce the risks existing in technological innovation activities of enterprises and the marginal cost of technological research and development of enterprises, thus improving the internal financing capacity of innovation activities [28]. It has a significant positive effect on the patent output of enterprises. But there are also views that financial subsidies will cause a "crowding out effect" on enterprise technological innovation [29].

The existing literature on public fiscal expenditure and industrial structure transformation and upgrading, as well as the relationship between them, is relatively rich, but there are still the following deficiencies. First of all, the evaluation of industrial transformation and upgrading is generally limited to the internal part of the three industrial structures, without considering the objective extension of industrial transformation and upgrading and total factor efficiency. A small number of studies using total factor productivity have not considered both non-radial and non-expected output due to the limitations of research methods. Secondly, the relevant research is mostly based on provincial-level data, while the research on urban level is relatively scarce. In addition, few scholars focus on the spatial impact of public fiscal expenditure and ignore the spatial spillover effect of public fiscal expenditure, so the role played by public fiscal expenditure in industrial transformation cannot be fully captured by them. Thirdly, whether public fiscal expenditures have an impact on industrial transformation remains a subject of debate, and the channels of their roles have not been clarified. Therefore, despite the importance of the previous literature for this study, some shortcomings should be addressed. Consequently, 250 Chinese cities' panel data from 2007 to 2020 is used to explore how public fiscal expenditure impacts industrial transformation and upgrading and put forward corresponding policy recommendations according to empirical results. It provides a theoretical and practical basis for promoting China's economic green transformation and ultimately achieving high-quality development.

## 3 Theoretical mechanism and research hypothesis

The government uses macro-control tools to ensure macroeconomic stability. As a key tool for macro-control, public fiscal expenditures play a significant role in industrial transformation and upgrading. According to Western economic thought, public fiscal expenditure will facilitate the development of a market economy through the multiplier effect, thus considerately promoting economic growth [30]. Especially, increasing public fiscal expenditures can improve the demand effectiveness and structure, thereby optimizing and upgrading industrial structure [31]. The public fiscal expenditure uses its guiding role and multiplier effect to affect the total social demand, enterprise investment income, and the way of resource allocation. The relatively loose budget expenditure system drives residents' consumption scale and diversification, which will guide the business direction, factor input planning, and decision-making of enterprises[11,32]. Adjusting the factor structure and flow, also guides enterprises to focus on improving the efficiency, circulation ability, and transformation quality of enterprise output factors. The expansion of scale directly affects the quantity and investment orientation of labor, capital, equipment, etc., promotes the aggregation and redistribution of industrial factors in various industrial sectors, further adjusts the composition of factors, and thus promotes the process of industrial transformation and upgrading [33]. Moreover, in parallel with the growth of the economy, human capital, and technology are constantly improving, which has laid a good external environment and social development environment for a country or region to upgrade its industrial structure. Industrial improvement is largely determined by the role fiscal expenditures play in national economic growth[34,35]. As economic development levels vary, the scale of public fiscal expenditures affects industrial transformation and upgrading differently. The increase in fiscal expenditure can realize the redistribution of resources, thus promoting the circulation and promotion of factors [36]. Therefore, a significant positive correlation exists between the scale of public fiscal expenditure and industrial upgrading and transformation [37,38]. However, public fiscal expenditure cannot increase indefinitely [39]. If it does so, it may produce a “crowding out effect”, suppressing relevant investment in enterprises [40,41], which is not conducive to stimulating enterprise vitality and thus inhibit industrial transformation and upgrading [42]. Additionally, it should be noted that under the background of the market economy, the excessive intervention of the government acting as the “night watchman” in industrial development will lead to negative effects such as “promotion tournament” of officials and “beggar-thy-neighbor” local protectionism [21] This triggers the convergence of regional industrial structure and brings adverse effects on regional industrial upgrading. In addition, in different periods of economic development, local government behaviors differ with the changes in industries and economic development stages [43]. Generally speaking, the more volatile the local government's fiscal behavior, the lower the proportion of industries with higher technical complexity [44,45], which significantly inhibits industrial transformation and upgrading. Conversely, an improvement in marketization will mitigate the negative effects of fluctuations in financial behavior [46]. As a result, local government behavior may significantly affect the industrial structure of the jurisdiction over the short term. And its leading effect decreases with the increase in the tenure of government officials, on the contrary, long-term trends depend mainly on market conditions [29].

To sum up, this study proposes Hypothesis 1: there may be an inverted U-shaped relation between the scale of public fiscal expenditure and industrial transformation and upgrading.

Having reviewed and summarized all available literature, this study assumes that public fiscal expenditure can affect industrial transformation and upgrading through three channels: technological innovation, resource dependence, and economies of scale.

(1) Technology innovation. The research, both at home and abroad, shows that technological innovation drives industrial development and transformation [2,47,48]. As technologies advance, backward production capacity and "black" industries can be withdrawn more quickly [49]. Public fiscal expenditure can enhance the enabling role of new technologies and accelerate the integrated development of new technology, new forms, and models of business through the implementation of strategic emerging industries [45]. With the government's plan to reduce the flow of production factors to low-end industries, more high-quality production factors will be directed to a more innovative green economy. In this way, the “tidal surge” is broken by stimulating investment diversification and upgrading, and realizing industrial transformation and upgrading. In addition, due to the encouragement and guidance of green consumption by public fiscal expenditure, the intensity of demand for low-end industrial capacity in the end-consumption market has been declining, forcing enterprises to take the initiative to adjust the production scale and product structure [37]. Expanding the supply of green products and services through technological innovation has become an important direction of enterprise strategy adjustment. As technological advancement pushes out low-end and outdated capacity, demand for high-end and green capacity increases. To achieve green transformation of enterprises, the government will invest more in new infrastructure construction, cooperate with the application of industrial policies, change market expectations, lead the direction of technology innovation and industrial reform, and thus promote industrial transformation and upgrading [33].
(2) Resource dependence. Many studies at home and abroad show that resource dependence will hinder industrial transformation and upgrading. In regions with rich resources, technology, and human capital are crowded out because of the "resource curse" [50], hindering industrial green transformation. The factor endowment and infrastructure in regions with rich resources are conducive to the agglomeration of resource-based industries, and high-tech industries are crowded out [9]. As a result, the black industries may flourish, which hinders the regional industrial transformation and upgrading. Additionally, while resource dependence can deliver great benefits in the short term, it will impair the creation of new driving forces, new industries, and new models for cities ultimately [51]. Due to the lack of advanced technology, high pollution, and high energy consumption are still prominent features of the black industry, leading to unsustainable development of the city. What's more, based on the “GDP” orientation of political performance assessment, local governments are inclined to invest in resource-based industries that can produce more economic benefits, however, these industries absorb and lock various production factors and prevent them from flowing, forming the simple industrial structure [52]. Facing such a situation, the market is unable to crack resource dependence, and government intervention is required [53]. As an important tool of macro-control, public fiscal expenditure is an effective way. Public fiscal expenditure supports economic green transformation. By reducing environmental protection tax and tax deductions, enterprises and individuals are encouraged to consume green, so as to reduce the negative effect of resource dependence on industrial transformation and upgrading.
(3) Scale economies. Scale economies will promote industrial transformation and upgrading. Following are some of the main ways. First, scale economies lower transportation costs between cities and transportation hubs as well as enterprise research and development costs [54,55]. On the other hand, scale economies enable enterprises to share public infrastructure, and reduce the shared cost of public resources, which is conducive to improving the transition of the value chain [56]. Second, scale economies enable enterprises in relevant industries to purchase new raw materials and professional services related to pollution treatment at a lower price, so that relevant industries can effectively deal with pollution emissions at a lower cost [26], thus realizing the green transformation and upgrading of the industry. The scale economies generated by industry agglomeration will also attract other industries, form diversified industrial clusters, provide multiple choices for related industries, and thus are more conducive to the adoption of clean technologies and environmental protection products in the industry. Resource sharing among enterprises not only reduces the related costs of enterprises, but also accelerates the green transformation of industries, reduces environmental pollution, and thus realizes industrial transformation and upgrading [57]. The public fiscal expenditure can effectively guide industrial agglomeration, form scale economies among industries, and then realize industrial transformation and upgrading.

**Fig. 2:**
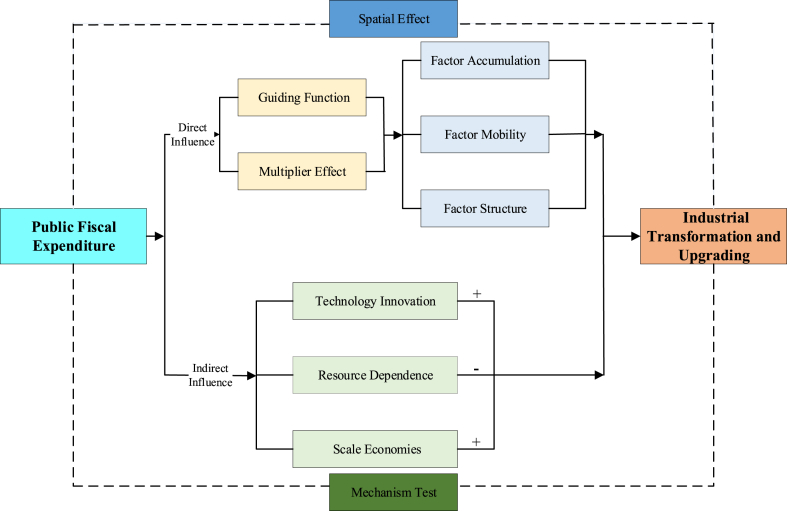
Research framework.

From the above theoretical analysis, we assume Hypothesis 2: Public fiscal expenditure indirectly leverages industrial transformation and upgrading through promoting technological innovation, reducing resource dependence, and expanding scale economies. Fig. 2 presents the Research framework of the paper.

## 4 Design of the study

### 4.1 Data source and variable description

A sample of 250 Chinese cities covering the period 2007–2020 is used for this study. Sources of original data include the China Urban Statistical Yearbook, China Statistical Yearbook, and the Financial Yearbook of China's Provinces, etc. Statistics of the variables are shown in Table 1.

(1) Explained variable Industrial transformation and upgrading (ITU). According to Ref. [58], the process of industrial transformation and upgrading involves continuously improving and evolving from low-end to high-end products, which not only includes the change of output value of the industrial sector but also improvements in the productivity of various industrial factors. Therefore, the calculation of the industrial structure transformation index is based on the super-efficient EBM model. The calculation equation using is as follows:

$$\gamma^{*}=\min_{\theta ,\lambda ,s^{-}}-\varepsilon_{x\sum_{i=1}^{m}\frac{\omega_{i}^{-}s_{i}^{-}}{x_{iq}}}$$

(1) $$s.t.\left\{ \begin{array}{l} \sum_{j=1}^{n}x_{ij}\lambda_{j}-\theta x_{iq}+s_{i}^{-}=0\;r=1,2,...,m\\ \sum_{j=1}^{n}y_{rj}\lambda_{j}\geq y_{rq\;}r=1,2,...,s\\ \lambda_{j}\geq 0,s_{i}^{-}\geq 0 \end{array}\right.$$

Wherein, $\gamma^{*}$ is the best effective value of the EBM measure DEA, θ represents the maximum effective value in the radial case, $s_{i}^{-}$ represents the maximum relaxation factor of the input factor i in the non-radial case, λ represents the relative weight of the input factor, $\left(x_{ij},y_{rj}\right)$ represents the maximum input-output vector of the DMU q, $\omega_{i}^{-}$ represents the relative weight of the type of input factor i, expresses the importance of the input factor i, and meets the requirements $\sum_{i=1}^{m}\omega_{i}^{-}=1$, $\varepsilon_{x}$ represents the core parameters that include both the radial change ratio and the non-radial relaxation vector, And meet $0\leq \varepsilon_{x}\leq 1$.

**Table 1:** Statistics of the variables.

Variable	Obs	Mean	Std. dev.	Min	Max
ITU	3500	2.4695	1.06503	0.2973	8.9639
PFESc	3500	0.8351	0.4457	0.3436	2.8128
ER	3500	3.3759	1.4124	0.2994	12.3890
*PD*	3500	5.7954	0.9098	1.5735	7.9226
FDL	3500	2.5698	1.3902	0.7091	16.6578
USC	3500	0.6485	1.4055	0.0152	43.2494
CAPITAL	3500	0.8530	0.3753	0.0319	2.9000

Given the complexity of the relationship between industrial transformation and upgrading, resources, and environment, including both desirable and undesirable outputs, as well as the existence of radial and non-radial contradictions, it is necessary to expand the super-efficient EBM model of equation (1) above into a non-radial EBM model based on undesirable outputs. The equation is as follows:

$$\gamma^{*}=\min\frac{\theta -\varepsilon_{x}\sum_{i=1}^{m}\frac{\omega_{i}^{-}s_{i}^{-}}{x_{iq}}}{\phi +\varepsilon_{y}\sum_{r=1}^{s}\frac{\omega_{r}^{+}s_{r}^{+}}{y_{rq}}+\varepsilon_{b}\sum_{p=1}^{u}\frac{\omega_{p}^{b-}s_{p}^{b-}}{y_{pq}}}$$

(2) $$s.t.\left\{ \begin{array}{l} \sum_{j=1}^{n}x_{ij}\lambda_{j}+s_{i}^{-}=\theta x_{iq}\;r=1,2,...,m\\ \sum_{j=1}^{n}x_{ij}y_{rj}-s_{r}^{+}=\phi y_{rq}\;r=1,2,...,m\\ \sum_{j=1}^{n}b_{pj}\lambda_{j}+s_{p}^{b-}=\phi b_{pq\;}p=1,2,...,u\\ \lambda_{j}\geq 0,s_{i}^{-}\geq 0,s_{r}^{+}\geq 0,s_{p}^{b-}\geq 0 \end{array}\right.$$

Wherein, $b_{pq}$ refers to the non-consensual output q of the city p; $\left(s_{r}^{+},s_{p}^{b-}\right)$ is the relaxation vector of the desirable output r and the undesirable output p; $\varepsilon_{y}$ and $\varepsilon_{b}$ are key parameters; ϕ indicates the output expansion ratio; $b_{pj}$ is the kind of undesirable output p of the DMU j; $b_{pq}$ represents the type of undesirable output p of the DMU q; u indicates the number of types of undesirable outputs. Other symbols have the same meanings as equation (1). Based on equations (1), (2), this study measures the level of industrial transformation and upgrading by using the super-efficiency EBM model of undesirable output.

**Fig. 3:**
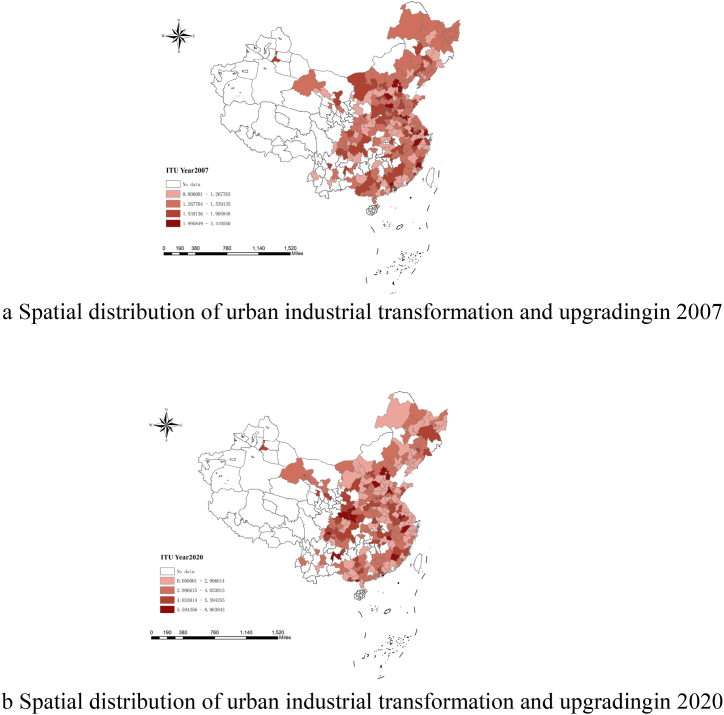
a Spatial distribution of urban industrial transformation and upgrading in 2007 Fig. 3-b Spatial distribution of urban industrial transformation and upgrading in 2020.

Drawing on existing research [3], this paper selects labor, capital stock, and total energy consumption as input factors, with the regional gross domestic product (NDP) of the three industries as the desired output, and wastewater, sulfur dioxide, and industrial smoke (powder) dust emissions generated in the industrial production process as the undesired output. Specifically, input elements include the following items: (1) Labor force. The total number of employees in the national economy of each city at the end of the year is used to measure labor input, with a unit of 10000 people. (2) Capital stock. This paper uses the "perpetual inventory method" to estimate capital stock. The formula is, $K_{t}=I_{t}+\left(1-\delta \right)K_{t-1}$. Where, $K_{t}$ is the capital stock in year t, $I_{t}$ is the investment in year t, and δ is the capital depreciation rate in year t. Here, based on the research results of Ref. [3], the value of δ is 9.6 %. The capital stock is all converted into the constant price in 2007 by the price index of fixed assets investment of the whole nation, and the unit is 100 million yuan. (3) Total energy consumption. This paper fully considers the structural differences and dynamic changes in energy consumption within the region and selects the energy consumption of various cities to measure energy input. Output elements involve the items as follows: ①The desired output. This paper takes 2007 as the base period and selects the three industries' Value Added (NDP) as the desirable output, with a unit of 100 million yuan. ②The undesired output. This paper uses the entropy method to calculate the scores of industrial wastewater, sulfur dioxide, and industrial smoke (powder) dust emissions in cities as undesirable output. We calculate the level of industrial transformation and upgrading in cities from 2007 to 2020 based on the above measurement methods. Spatial distributions of urban industrial transformation and upgrading in 2007 and 2020 are shown in Fig. 3-a and Fig. 3-b.

As presented in Fig. 3-a and Fig. 3-b, the study period saw a substantial improvement in industrial transformation and upgrading, demonstrating obvious spatial agglomeration characteristics. Industrial transformation and upgrading in central and western regions is relatively fast, but the development trend is balanced on the whole, which may benefit from the combined effects of several policies, involving China's fiscal policy, and the trend will be more optimized as the effects of policies are released.

(2) Explanatory variables

Public Fiscal Expenditure (PFESc) is characterized by the proportion of public fiscal expenditure and GDP [11].

(3) Control variable

Capital level (CAPITAL) This paper mainly uses the proportion of fixed asset investment in GDP to reflect the capital level [59]. Financial Development Level (FDL) It is measured by the ratio of year-end deposit and loan balance to GDP in each city [60]. Urban Spiritual Civilization Level (USC) It reflects the environmental awareness of urban residents and affects the green level of production and life. Therefore, it is reflected by the per capita collection of books in each city each year [61]. Environmental regulation (ER) It is measured by the proportion of the word frequency related to the word “environmental protection” in the work report [62]. Population Density (PD) It is reflected by 1000 people per square kilometer [6].

### 4.2 Measurement model setting

Cities are interdependent communities of life. Industrial transformation and upgrading, as an important exogenous factor in urban economic development, may form spatial interaction of industrial structure with resource factors flowing and traffic improvement [53]. Therefore, a spatial econometric model is adopted to examine how public fiscal expenditures affect industrial transformation and upgrading.

Additionally, a quadratic term of public fiscal expenditure is included to account for the potential nonlinearity between them. As shown in equation (3), we construct the spatial measurement model as follows:

(3) $$\begin{array}{l} \left\{ \begin{array}{l} ITU_{it}=\alpha +\rho \sum_{j=1}^{N}W_{ij}ITU_{it}+\beta_{1}PEFS_{it}+\beta_{2}PEFS_{it}^{2}\\ +\beta_{3\sum_{n=1}^{5}X_{it}}+\theta_{1}W_{ij}PEFS_{it}+\theta_{2}W_{ij}PEFS_{it}^{2}+\theta_{3\sum_{n=1}^{5}W_{ij}X_{it}}+u_{i}+\lambda_{t}+\varepsilon_{it} \end{array}\right. \end{array}$$

Where i represents the city, t represents the year, $ITU_{it}$ represents the industrial transformation and upgrading index of the city i in the period t, $PEFS_{it}$ represents the scale of public fiscal expenditure, $X_{it}$ is a group of control variables, $u_{i}$ represents individual effect, $\lambda_{t}$ represents time effect, and $\varepsilon_{it}$ is a random disturbance term, $W_{ij}$ is a spatial weight matrix element. The reciprocal square weight matrix of distance is adopted in this paper for the setting of the spatial weight matrix, which can capture spatial associations between cities more accurately. According to the research [11,53], the nested weight matrix of economic geography is established. Equation (4) is a specific setting method:

$$W_{ij}^{e}=W_{ij}^{d}\times diag\left(\overset{-}{E}/\overset{-}{E},\overset{-}{E_{2}}/\overset{-}{E},\cdot \cdot \cdot \cdot \cdot \cdot \overset{-}{E_{n}}/\overset{-}{E}\right)$$

(4) $$W_{ij}^{e}=\left\{ \begin{array}{l} \frac{W_{ij}^{e}}{\sum_{i}W_{ij}^{e}},i\neq j\\ 0,i=j \end{array}\right.$$

Where, $\overset{-}{E_{i}}=1/\left(t_{1}-t_{0}+1\right)\sum_{t_{0}}^{t_{1}}E_{ij}$ is the average per capita GDP of the city i, $\overset{-}{E}=1/\left(t_{1}-t_{0}+1\right)\sum_{i=0}^{n}\sum_{t_{0}}^{t_{1}}E_{ij}$ is the average per capita GDP of all cities, and t is in different periods. The above matrix shows that when a city's per capita GDP accounts for a large proportion of the total (i.e. $\overset{-}{E_{i}}/\overset{-}{E}>\overset{-}{E_{j}}/\overset{-}{E}$), a greater impact is also felt in the surrounding areas (i.e. $W_{ij}>W_{ji}$).

## 5 Empirical analysis and discussion

### 5.1 Empirical analysis

**Fig. 4:**
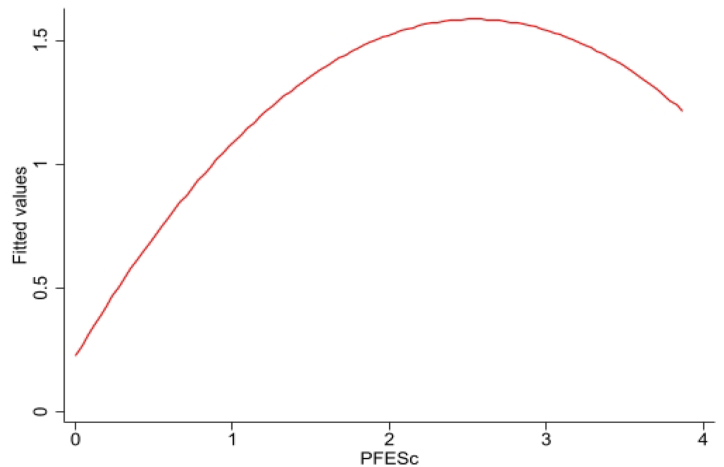
A fitting map of public financial expenditure and industrial structure transformation and upgrading from 2007 to 2020.

Theoretical analysis suggests a potential nonlinear correlation between public financial spending and industrial transformation and upgrading. To preliminarily verify this relationship, we used Stata software to fit the sample data from 2007 to 2020. Fig. 4 shows that a "U" curve is evident between them, which indicates that with the increase of public financial expenditure, its promotion effect gradually increases, and after reaching the "peak", the effect starts to go away. This result provides a basis for the following empirical test.

#### 5.1.1 Spatial correlation analysis

**Fig. 5:**
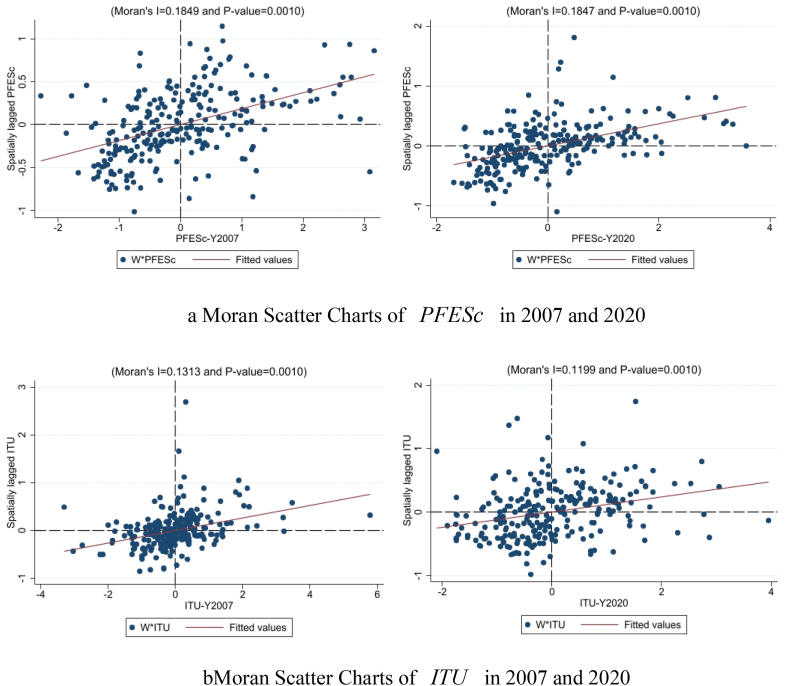
A Moran scatter charts of PFESc in 2007 and 2020 Fig. 5-b Moran scatter charts of ITU in 2007 and 2020.

Testing spatial correlations between variables is done with a Moran's I test. The significance test in Table 2 shows that both PFESc and ITU pass from 2007 to 2020, suggesting a spatial relationship seems to exist. Moran scatter diagrams are presented in Fig. 5-a and Fig. 5-b to depict the spatial agglomeration in 2007 and 2020.

**Table 2:** Moran'I index of PFESc and ITU in 2007–2020.

Year	PFESc	ITU	Year	PFESc	ITU
2007	0.1850∗∗∗	0.1310∗∗∗	2014	0.1910∗∗∗	0.2130∗∗∗
2008	0.2710∗∗∗	0.1290∗∗∗	2015	0.2010∗∗∗	0.2030∗∗∗
2009	0.2120∗∗∗	0.1170∗∗∗	2016	0.0890∗∗∗	0.2270∗∗∗
2010	0.1990∗∗∗	0.1330∗∗∗	2017	0.1950∗∗∗	0.1690∗∗∗
2011	0.2380∗∗∗	0.1570∗∗∗	2018	0.1880∗∗∗	0.1570∗∗∗
2012	0.2320∗∗∗	0.1750∗∗∗	2019	0.1830∗∗∗	0.1460∗∗∗
2013	0.2010∗∗∗	0.2010∗∗∗	2020	0.1850∗∗∗	0.1200∗∗∗

#### 5.1.2 Model selection for spatial econometrics

As a preliminary step, we perform a test of model applicability. Tests of the spatial econometric model confirm their suitability for empirical studies as shown in Table 3. For more information on the specific test principles and process, it is available in the research [53].

**Table 3:** Selection of spatial measurement model.

Index	Value	P-value	Index	Value	P-value
LM-lag	38.16	0.000	LM-error	42.98	0.000
Robust LM-lag	157.85	0.000	Robust LM-error	191.64	0.000
LR-lag	22.92	0.013	LR-error	21.89	0.013
WALD-SAR	11.98	0.0009	WALD-SEM	10.89	0.0010
Hausman	16.11	0.013			

#### 5.1.3 Baseline regressions

An empirical test is undertaken using a dynamic SDM in response to the test of model applicability. Table 4 shows the test results. The spatial autoregressive coefficient (ρ) and spatial autocorrelation coefficient (λ) in each model are positively significant, which indicates that the spatial interaction effect exists, and also verifies that using spatial econometric models is a more reasonable way to explore the impact of public expenditure on industrial transformation and upgrading.

**Table 4:** Baseline regression results.

Variables	(1)	(2)	(3)	(4)
	Sys-GMM	SAR	SEM	SDM
PFESc	0.0708∗∗∗	0.0131∗∗∗	0.0141∗∗∗	0.0166∗∗∗
	(0.0101)	(0.0032)	(0.0033)	(0.0034)
$PFESc^{2}$	−0.0002∗∗	−0.0001∗∗∗	−0.0001∗∗∗	−0.0001∗∗∗
	(0.0001)	(0.00002)	(0.00004)	(0.00001)
ER	0.0404∗∗∗	0.0092	0.0065	0.0028
	(0.0105)	(0.0067)	(0.0068)	(0.0067)
PD	−0.0007∗∗∗	0.0005∗∗∗	0.0007∗∗∗	0.0008∗∗∗
	(0.0002)	(0.0002)	(0.0002)	(0.0002)
FDL	0.1004∗∗∗	0.0279∗	0.0280∗	0.0190
	(0.0220)	(0.0152)	(0.0156)	(0.0152)
USC	−0.0804∗∗∗	0.0070	0.0081	0.0037
	(0.0283)	(0.0069)	(0.0068)	(0.0069)
CAPITAL	2.5607∗∗∗	0.5190∗∗∗	0.5090∗∗∗	0.3360∗∗∗
	(0.1603)	(0.0428)	(0.0482)	(0.0470)
W∗PFESc				−0.0153
				(0.0104)
AR(1)	−2.19[0.029]			
AR(2)	−0.46[0.642]			
Hansen	11.92[0.452]			
ρ		0.3360∗∗∗		0.2220∗∗∗
		(0.0322)		(0.0343)
λ			0.3150∗∗∗	
			(0.0365)	
sigma2_e		0.1730∗∗∗	0.1750∗∗∗	0.1650∗∗∗
		(0.0043)	(0.0044)	(0.0041)
Year-fixed	Y	Y	Y	Y
City-fixed	Y	Y	Y	Y
*Log-likelihood*		8042.9	8045.9	8049.7
*N*	3500	3500	3500	3500

Note: ∗∗∗, ∗∗, and ∗ are significant at the level of 1 %, 5 %, and 10 % respectively, within ( ) are the standard error values, and [ ] are P values. The following are the same.

The coefficient of the core explanatory variable PFESc is significantly and positively related to industrial transformation and upgrading whether in the non-spatial econometric model or the spatial econometric model, indicating that urban industrial transformation and upgrading will be enhanced as public fiscal expenditure increases. The coefficient of $PFESc^{2}$ is significantly negative, verifying the research hypothesis H1, that is, there exists an inverted U-shaped relation between public fiscal expenditure and industrial transformation and upgrading.

Population density (PD) and fixed capital investment (CAPITAL) are significantly positive, indicating that the local population has formed effective human capital. As an essential component of production, human capital may greatly improve the quality of human capital due to factors such as education level, thus contributing to industrial upgrading [48]; The combination of capital and labor, namely capital deepening, will also promote the transformation of industrial structure from capital-intensive sectors to labor-intensive sectors. Although the environmental regulation (ER), financial development level (FDL), and spiritual civilization construction level (USC) are positive, indicating that industrial transformation and upgrading are only marginally impacted by them, and there is still much room for development.

#### 5.1.4 Heterogeneity analysis

In this study, the heterogeneous impact of public fiscal expenditure on industrial transformation and upgrading is further examined from the perspective of urban scale. As stated in the provisions of the Notice of the State Council in which division is based on the resident population of each city from the last year of the study period to 2020, the samples are divided into 15 megacity behemoths, 79 megacities, 149 large-sized cities, and 7 small and medium-sized cities. Estimated results are shown in Table 5.

**Table 5:** Estimated results of heterogeneity test.

Variables	Small and medium-sized cities	Large-sized cities	Megacity	Megacity behemoths
	(1)	(2)	(3)	(4)
PFESc	0.1037∗∗∗	0.0180∗∗∗	0.0204∗	−0.0058∗∗
	(0.0307)	(0.0045)	(0.0115)	(0.0025)
$PFESc^{2}$	−0.0011∗∗∗	−0.0001∗∗∗	0.0005∗∗	−0.0006∗∗
	(0.0003)	(0.0000)	(0.0002)	(0.0003)
W∗PFESc	0.0325	−0.0385∗∗∗	0.0393	0.4046∗∗∗
	(0.0456)	(0.0128)	(0.0348)	(0.0803)
Control	Y	Y	Y	Y
ρ	−0.0880∗∗	0.3040∗∗∗	−0.1957∗∗∗	−0.3929∗∗∗
	(0.0446)	(0.0404)	(0.0534)	(0.1086)
sigma2_e	0.0875∗∗∗	0.1815∗∗∗	0.0937∗∗∗	0.0430∗∗∗
	(0.0131)	(0.0059)	(0.0042)	(0.0044)
Year-fixed	Y	Y	Y	Y
City-fixed	Y	Y	Y	Y
*N*	98	2086	1016	210

#### 5.1.5 Robustness analysis

A robustness test is conducted in this paper using the following methods: (1) Remeasuring the explained variables with the method [63]. $ISU=\sum_{i=1}^{3}\frac{P_{i}}{P}LE_{i}$. $P_{i}$ and P respectively represent the output value of the industry i and the gross domestic product of the city, $LE_{i}$ is the labor productivity of the industry i. (2) Replacing the spatial weight with the inverse distance spatial weight matrix. (3) Excluding the four municipalities^1^ from the samples. These regressions shown in Table 6 give similar results to the benchmark regression, proving its credibility.

**Table 6:** Robustness test result.

Variables	Recalculate the explained variables	Replace the spatial weight	Exclude the four municipalities
	(1)	(2)	(3)
PFESc	1.2370∗∗∗	0.0142∗∗∗	0.0170∗∗∗
	(0.3437)	(0.0033)	(0.0034)
$PFESc^{2}$	−0.0489∗	−0.0001∗∗∗	−0.0001∗∗∗
	(0.0279)	(0.00002)	(0.00003)
W∗PFESc	0.2570	0.1765∗	−0.0210∗∗
	(0.6946)	(0.0987)	(0.0103)
Control	Yes	Yes	Yes
ρ	0.2114∗∗∗	1.1704∗∗∗	0.2007∗∗∗
	(0.0390)	(0.2598)	(0.0345)
sigma2_e	10.7670∗∗∗	0.1687∗∗∗	0.1631∗∗∗
	(0.2678)	(0.0042)	(0.0041)
Year-fixed	Y	Y	Y
City-fixed	Y	Y	Y
*N*	3500	3500	3444

#### 5.1.6 Mechanism analysis

To further identify how public fiscal expenditure impacts industrial transformation and upgrading, based on Ref. [64], we construct three regression equations for tests. We speculate that public fiscal expenditure indirectly impacts industrial transformation and upgrading through technological innovation, resource dependence, and scale economies. R&D personnel are a measure of technological innovation(INNOV); Resource dependence (EXTIND) is reflected by the number of extractive workers; Scale economies(GDP)is measured by urban GDP.

(5) $$ITU_{it}=\alpha_{0}+\alpha_{1}PFES+\alpha_{2}X_{it}+\mu_{it}$$

(6) $$M_{it}=\beta_{0}+\beta_{1}PFES+\beta_{2}X_{it}+\mu_{it}$$

(7) $$ITU_{it}=\lambda_{0}+\lambda_{1}PFES+\lambda_{2}M_{it}+\lambda_{3}X_{it}+\mu_{it}$$

There are several control variables arranged in a vector X; M is a mediation variable.

The estimations based on a double fixed effect model are presented in Table 7. According to the intermediary effect test rule [65], if $\alpha_{1}$ in equation (5), $\beta_{1}$ in equation (6), and $\lambda_{1}$ in equation (7) are all significant, it indicates that an intermediary effect is evident. Specifically, Columns (1)–(3) present the intermediary effect test of technological innovation in which the total effect of public fiscal expenditure on industrial transformation and upgrading is 0.01142. Column (2) shows the coefficient of technological innovation is significantly positive at the level of 1 %, indicating that public fiscal expenditure has significantly promoted technological innovation. Column (3) shows that industrial transformation and upgrading are affected by technological innovation and public fiscal expenditure. The coefficients are all strongly positive, and the coefficient of public fiscal expenditure (0.01136) in equation (7) is less than that (0.01142) in equation (5), indicating that public fiscal expenditure not only directly affects industrial transformation and upgrading, but also indirectly promotes urban industrial transformation and upgrading by promoting urban technological innovation. Moreover, there is significant significance at the level of 1 % in both the Sobel and Bootstrap tests, which also confirm that a significant intermediary effect exists, with a ratio of 7.60 % of intermediary effect to the total effect. A similar analysis shows that 4.25 % and 10.78 % of the total effects are attributed to resource dependence and scale economies, respectively. As a result, H2 is verified. Analyzing the three intermediary effects, we find that scale economies are the largest, followed by technological innovation and resource dependence.

**Table 7:** Mechanism test results.

	(1)	(2)		(3)	(4)	(5)	(6)	(7)
Intermediary Effect		Technological innovation			Resource dependence		Scale economies	
Explained Variable	ITU	INNOV		ITU	EXTIND	ITU	GDP	ITU
PFESc	0.01142∗∗	0.03202∗∗∗		0.01136∗∗∗	−0.0418∗∗∗	0.0109∗	0.0067∗∗∗	0.0102∗∗
	(0.0063)	(0.0179)		(0.0063)	(0.0664)	(0.0063)	(0.0014)	(0.0106)
M				0.00190∗∗		−0.0116∗∗∗		0.1838∗∗
				(0.0156)		(0.0017)		(0.0937)
Sobel		0.0001 (Z = 1.79,p = 0.0720)			0.0005 (Z = 1.8012,p = 0.0717)		−0.0012 (Z = −4.7568,p = 0000)	
	
Indirect Effect Proportion		7.60 %			4.25 %		10.78 %	
*R^2^*	0.3016	0.2720		0.3016	0.1666	0.2875	0.4556	0.2780

Control	Y	Y		Y	Y	Y	Y	Y
Year-fixed	Y	Y		Y	Y	Y	Y	Y
City-fixed	Y	Y		Y	Y	Y	Y	Y

### 5.2 Discussion of study results

#### 5.2.1 Discussion of the baseline regression results

Table 4 shows that urban industrial transformation and upgrading will be enhanced as public fiscal expenditure increases. The increase of public fiscal expenditure promotes industrial transformation and upgrading at the primary stage, and then inhibits it after reaching the peak, which implies that there is an optimal scale of public fiscal expenditure. The scale of public fiscal expenditure influences social demand through its guidance and incentive multiplier effect, guides the business direction, factor input plan, and investment decision of enterprises, and thus promotes the process of industrial transformation and upgrading [11]. Specifically, from the perspective of demand, the scale of social demand will directly affect the industrial scale, and then affect the industrial transformation and upgrading. Government fiscal expenditures can be adjusted with policy tools such as contingent decisions and automatic stabilizers to suit the effective social demand and move the budget in an environmentally friendly and greener direction [55]. Public fiscal expenditure can guide the change of consumption demand, investment demand, and export demand, make the demand structure more reasonable, trigger the advanced rationalization of the structure of consumer goods, investment goods, and export products, and thus drive industrial transformation and upgrading. From the supply side, the scale of public fiscal expenditure, as an industrial guidance signal sent by the government, can guide social funds and other factor resources to develop strategic emerging industries and high-tech industries and encourage enterprises to adopt economic strategies that conform to the direction of social development. A well-allocated labor force, capital, technology, and other factors are crucial to industrial development [66]. As a result, public fiscal expenditures are crucial to industrial transformation and upgrading. However, if the government intervenes too much, "promotion tournaments" will be held among officials and a "beggar-thy-neighbor" mentality will develop. In turn, regional industrial structures will be converged, which will adversely affect their upgrading [63].

#### 5.2.2 Discussion of the heterogeneity test results

Table 5 shows that fiscal expenditures have a decreasing effect on the transformation and upgrading of industrial structures as cities expand. Possibly, public fiscal expenditures account for a relatively large share of industrial inputs in small and medium-sized cities, therefore, public fiscal expenditure has a more obvious impact on industrial transformation and upgrading [67]. Comparatively, public fiscal expenditure in megacity behemoths cannot meet the needs of urban-scale development, the input-output ratio is not reasonable, and scientific and technological achievements fail to be converted into real productivity, thus inhibiting the transformation and upgrading of industrial structure.

#### 5.2.3 Discussion of the mechanism test results

As shown in Table 7, public fiscal expenditure not only directly affects industrial transformation and upgrading, but also indirectly leverages industrial transformation and upgrading through promoting technological innovation, reducing resource dependence, and expanding scale economies. Analyzing the three intermediary effects, we find that scale economies are the largest, followed by technological innovation and resource dependence. The reason is that scale economies can enhance the competitiveness of enterprises and provide necessary conditions for industrial transformation and upgrading [42]. Especially, urban factor resources can be allocated and used more efficiently as a result of it and provide more space for public fiscal expenditure for local governments to cultivate emerging industries, so as to promote transformation and upgrading. With public fiscal expenditures, enterprises can focus on high-tech research and development, and thus industrial transformation and upgrading are achieved [68]. In addition, resource dependence may form a “resource curse” and inhibit urban industrial transformation and upgrading. However, resource dependence can be alleviated by increasing public fiscal expenditures to support the development of medium- and high-end industrial technologies, which may be the reason for the weaker effect of resource dependence on industrial transformation and upgrading [69,70].

## 6 Conclusions and policy recommendations

### 6.1 Conclusions

This paper empirically investigates how public fiscal expenditure impacts industrial transformation and upgrading by applying spatial econometric models and mechanism testing models based on the data of 250 Chinese cities from 2007 to 2020. It provides a theoretical and practical basis for promoting China's economic green transformation and ultimately achieving high-quality development. There are three main findings: (1) Public fiscal expenditure serves a critical role in facilitating industrial transformation and upgrading, but their relationship resembles an inverted U. Therefore, an optimal scale of public fiscal expenditure exists. (2) Heterogeneity findings suggest that the promoting effect of public fiscal expenditure on the industrial transformation and upgrading decreases with the expansion of the city scale. (3) The role mechanism implies that public fiscal expenditure indirectly leverages industrial transformation and upgrading through promoting technological innovation, reducing resource dependence, and expanding scale economies. Among the three channels, the effect exerted by scale economies is the largest, followed by technological innovation and resource dependence.

### 6.2 Policy recommendations

Based on the findings above, the policies are suggested as follows.

(1) Establish and improve the dynamic monitoring system of the scale of public fiscal expenditure. Specially, construct and improve the monitoring and early warning system of industrial transformation and upgrading on a national level, and implement scientific control over the scale of public fiscal expenditure. More importantly, the implementation effect of the monitoring and early warning system should be included in the official political performance assessment system. Meanwhile, guiding suggestions on the scale of public fiscal expenditure according to different city sizes should be proposed. Depending on the local conditions, regional governments should continuously increase public fiscal expenditure before the maximum is reached. In particular, utilize funds to develop new industries and tertiary industries, so that the multiplier effect and guiding role of public expenditures are fully realized, and ultimately better serve the industrial transformation and upgrading.
(2) Build a cross-regional coordination mechanism to continuously narrow the inter-regional industrial transformation and upgrading gap. China is vast in territory and different in resource endowments across regions, so it is crucial to identify the "key factors" causing these differences. Following the key factors, different public expenditure policies should be adopted in different cities and one-size-fits-all policies are not advisable. In addition, cross-regional collaborative regulatory platforms should be established to maximize the spatial positive spillover effects of technologies and policies, enhance technological innovation of cities in less developed areas, and gradually narrow the inter-regional industrial transformation and upgrading level gap utilizing matching support and strengthening East-west cooperation.
(3) Maximize the contribution of public fiscal expenditure in industrial transformation and upgrading. Investment in technological innovation should be increased, especially enterprises' R&D in low-carbon and clean technologies in key fields should be enhanced. More importantly, public fiscal expenditure should support emerging industries, reduce the dependence on traditional energy, constantly break the low-end lock of resource dependence and low-level industrial development, foster emerging industries and enterprises, and increase the R&D of new energy and its application, completely get rid of the "black and gray" industry fetters. In addition, public fiscal expenditure should also promote the formation of strategic emerging industrial clusters, constantly extend the industrial chain, form scale economies, and ultimately promote industrial transformation and upgrading.

There are many channels through which public fiscal expenditure indirectly affects industrial transformation and upgrading. As a result of the unavailability of data, only three channels are studied in this paper. In future research, the theoretical analysis and empirical test should incorporate more channels. Moreover, most of the existing literature adopts mathematical models and empirical tests to discuss how to improve industrial transformation and upgrading. It is a feasible way to expand the theory and systematically summarize experience through case studies to further improve relevant studies.

## Data availability statement

Data will be made available on request.

## CRediT authorship contribution statement

**Junfeng Zhao:** Writing – review & editing, Writing – original draft, Funding acquisition, Formal analysis, Data curation, Conceptualization. **Jinling Yan:** Supervision, Software, Project administration, Funding acquisition.

## Declaration of competing interest

The authors declare the following financial interests/personal relationships which may be considered as potential competing interests:Junfeng Zhao reports financial support was provided by Tianchi Talents Innovation Leading Talent Project (40120001). Junfeng Zhao reports financial support was provided by Postgraduate Education and Teaching Reform Research Project of 10.13039/501100008526Xinjiang University of Finance and Economics in 2024 (XJUFE2024YJG05). Jinling Yan reports financial support was provided by Research Funds for Universities of Xinjiang Autonomous Region (XJEDU2024J102). If there are other authors, they declare that they have no known competing financial interests or personal relationships that could have appeared to influence the work reported in this paper.

## Footnotes

1 The four municipalities include Beijing, Tianjin, Shanghai, and Chongqing in China.
